# Enterohepatic Takeda G-Protein Coupled Receptor 5 Agonism in Metabolic Dysfunction-Associated Fatty Liver Disease and Related Glucose Dysmetabolism

**DOI:** 10.3390/nu14132707

**Published:** 2022-06-29

**Authors:** Justine Gillard, Corinne Picalausa, Christoph Ullmer, Luciano Adorini, Bart Staels, Anne Tailleux, Isabelle A. Leclercq

**Affiliations:** 1 Laboratory of Hepato-Gastroenterology, Institute of Experimental and Clinical Research, Université Catholique de Louvain, 1200 Brussels, Belgium; justine.gillard@uclouvain.be (J.G.); corinne.picalausa@student.uclouvain.be (C.P.); 2 Pharma Research & Early Development, Roche Innovation Center Basel, F. Hoffmann-La Roche Ltd., 4070 Basel, Switzerland; christoph.ullmer@roche.com; 3 Intercept Pharmaceuticals, New York, NY 10001, USA; ladorini@interceptpharma.com; 4 Inserm, CHU Lille, Institut Pasteur de Lille, University Lille, U1011-EGID, F-59000 Lille, France; bart.staels@pasteur-lille.fr (B.S.); anne.tailleux@univ-lille.fr (A.T.)

**Keywords:** MAFLD, NASH, metabolic syndrome, TGR5, INT-777, RO5527239, FXR, fexaramine

## Abstract

Metabolic dysfunction-associated fatty liver disease (MAFLD) is a major health concern with no approved pharmacological therapies. Molecules developed to activate the bile acid-receptor TGR5 regulate pathways involved in MALFD pathogenesis, but the therapeutic value of TGR5 activation on the active form of MAFLD, non-alcoholic steatohepatitis (NASH), still needs to be evaluated. As TGR5 agonism is low in MAFLD, we used strategies to promote the production of endogenous TGR5 ligands or administered pharmacological TGR5 agonists, INT-777 and RO5527239, to study the effect of TGR5 activation on liver and metabolic diseases in high-fat diet-fed *foz/foz* mice. Although described in the literature, treatment with fexaramine, an intestine-restricted FXR agonist, did not raise the concentrations of TGR5 ligands nor modulate TGR5 signaling and, accordingly, did not improve dysmetabolic status. INT-777 and RO5527239 directly activated TGR5. INT-777 only increased the TGR5 activation capacity of the portal blood; RO5527239 also amplified the TGR5 activation capacity of systemic blood. Both molecules improved glucose tolerance. In spite of the TGR5 activation capacity, INT-777, but not RO5527239, reduced liver disease severity. In conclusion, TGR5 activation in enterohepatic, rather than in peripheral, tissues has beneficial effects on glucose tolerance and MAFLD.

## 1. Introduction

Metabolic dysfunction-associated fatty liver disease (MAFLD) ranges from benign hepatic steatosis to non-alcoholic steatohepatitis (NASH), which is characterized at histology by steatosis, lobular inflammation and hepatocyte ballooning, with variable degrees of fibrosis [1]. Further worsening of disease activity and fibrosis leads to cirrhosis and hepatocellular carcinoma [2]. To clarify diagnosis criteria, ‘NAFLD’ for non-alcoholic fatty liver disease is being replaced by ‘MAFLD’ for metabolic dysfunction-associated fatty liver disease. This emphasizes the tight link among liver disease, metabolic syndrome and cardiovascular risk [3,4]. While increasingly common and representing a major health concern, MAFLD remains currently with no approved pharmacological therapies, also because our understanding of the disease is incomplete [2,5].

During this last decade, two bile acids receptors, namely Farnesoid X receptor (FXR) and Takeda G-protein coupled receptor 5 (TGR5), have been under the spotlight as targets for the treatment of MAFLD. Indeed, their activation by natural bile acids or by synthetic agonists controls pathways that are disrupted in MAFLD, such as lipid and glucose homeostasis [6,7,8,9,10], inflammation [11,12,13,14] and fibrogenesis [15].

Several TGR5 agonists have been developed and studied in models of metabolic syndrome. The synthetic cholic acid-derivate INT-777 improved insulin sensitivity and hepatic steatosis in mouse models [6]. The synthetic ursodeoxycholic acid-derivate BAR501 also increased insulin sensitivity and reduced hepatic steatosis, inflammation and fibrosis in mice with mild steatohepatitis [16,17]. The non-steroidal RO5527239 increased glucagon-like peptide 1 (GLP-1) secretion from the enteroendocrine L cells and thereby enhanced glucose tolerance [18,19]. In addition, a shift in gut microbiota has been shown to increase the endogenous production of bile acids with high affinity for TGR5 [20]. This represents another tool to increase TGR5 activation. To illustrate, fexaramine (FEX), a synthetic intestine-restricted FXR agonist, shaped the gut microbiota, resulting in increased synthesis of secondary bile acids, stimulation of TGR5 signaling and improvement of glucose metabolism [20,21]. All the above support a beneficial effect of increased TGR5 activation, but its therapeutic effect on NASH has not yet been tested. 

In a previous study, we demonstrated the contribution of bile acids to the development of NASH in two different validated mouse models [22]. The enterohepatic perturbation of bile acids resulted in reduced signaling via TGR5 and FXR in mice with NASH; and the restoration of TGR5 and FXR signaling by deoxycholic acid (DCA) supplementation protected from NASH and associated metabolic features [22]. DCA is a strong TGR5 agonist (EC_50_ = 1.01–1.25 µM), but it also activates FXR (EC_50_ = 50–75 µM) [23]. It is therefore uncertain whether the observed effects depend on the engagement of TGR5 and/or of FXR. 

Hence, our aim was to identify whether TGR5 agonists are hepatoprotective in mice with active MAFLD. We used specific pharmacological TGR5 agonists, INT-777 and RO5527239, or strategies to promote the production of endogenous TGR5 agonists. 

## 2. Materials and Methods

### 2.1. Animals and Treatments

Animal care was provided in accordance to the guidelines for humane care for laboratory animals as per the European regulations. The study protocol was approved by the university ethics committee for the use of experimental animals under the reference 2020/UCL/MD/018. 

We used male *foz/foz* (*Alsm1* ^−/−^) and WT (*Alms1* ^+/+^) mice on a NOD.B10 background [24,25]. When fed a high-fat diet (HFD, Research Diets D12492), *foz/foz* mice develop obesity, insulin resistance and steatohepatitis, while WT mice have moderate metabolic alterations but no liver disease [26]. 

In a first experiment, six-week-old *foz/foz* mice were fed an HFD for 8 weeks, then divided in two groups based on their body weight and glycemia (n = 11–13/group). One group received an HFD and the other one received an HFD supplemented with fexaramine (FEX, MedChemExpress) for an additional 3 weeks. The concentration of FEX in the diet was adjusted according to food intake for the mice to receive 50 mg per kg of body weight daily.

In a second experiment, six-week-old *foz/foz* mice and WT mice were fed an HFD for 4 weeks (n = 6–7/group). Then, *foz/foz* mice were divided in three groups based on their body weight and glycemia and received an HFD, an HFD containing INT-777 (a gift from Intercept Pharmaceuticals [6]) or an HFD containing RO5527239 (a gift from F. Hoffmann-La Roche Ltd. [18]) for one additional week. The concentration of the compounds in the diet was adapted for the mice to receive 30 mg of INT-777 or 10 mg of RO5527239 per kg of body weight daily. WT mice were kept under an HFD for the whole experiment. 

Alternatively, seven-week-old *foz/foz* mice were divided into three groups based on their body weight and glycemia and received an HFD alone, an HFD containing INT-777 or RO5527239 for 12 weeks (n = 6–7/group). They were compared to WT mice fed an HFD for 12 weeks (n = 7).

Body weight, glycemia and food intake were measured once a week. Before sacrifice, mice were fasted for 12 h; then, they were refed for 4 h to synchronize for intestinal bile secretion and anesthetized with ketamine–xylazine. Portal and cardiac blood were collected in heparin-coated tubes and plasma stored at −80 °C. Liver, distal ileum, colon and brown adipose tissue were harvested, weighted, snap frozen in liquid nitrogen and stored at −80 °C or fixed in 4% formalin. 

### 2.2. Oral Glucose Tolerance Test (OGTT) and Serum Tests

After 4 h of fasting, mice were administered 40 mg of glucose by oral gavage. Blood glucose was measured at 0, 15, 30, 60, 90, 120 and 180 min after gavage using a glucometer. Total GLP-1 concentration was measured in serum harvested during the OGTT 15 min after the glucose administration, in a tube containing a DPPIV inhibitor (Merck, DPP4-010), using a Meso Scale Discovery assay (C0292-2). Active GLP-1 concentration was measured in serum harvested during the OGTT 30 min after the glucose administration in a tube containing a DPPIV inhibitor (Merck, DPP4-010), using a Merck ELISA (EGLP-35K). Insulin concentration was measured in serum harvested in 4 h-fasted mice, using an Ultrasensitive Mouse Insulin ELISA kit (Mercodia, Uppsala, Sweden, 10-1249-01). Plasmatic concentrations of alanine aminotransferase (ALT) were measured using a DRI-CHEM NX500. 

### 2.3. Liver Density

The ratio of liver-to-spleen density, a surrogate for liver steatosis, was assessed by microcomputed tomography as previously described [26]. 

### 2.4. TGR5 Reporter Assay

TGR5 ligand activity was measured in a cell reporter assay as previously described and validated [22]. Briefly, HEK293T cells (ATCC CRL-3216) were cultured in DMEM containing 10% FBS and 1% Penicillin/Streptomycin. At 80% of confluence in a 96-well plate, cells were transfected with 20 ng of pCMV-SPORT6 human GPBAR1 (Harvard Medical School MGC:40597), 40 ng of pGL4.29 (CRE-luciferase, Promega, Madison, USA) and 5 ng of pGL4.73 (SV40-Renilla, Promega) using Lipofectamine 2000. Twenty-four hours later, cells were incubated with FBS-free medium (CTL) or FBS-free medium containing tauro-lithocholic acid (TLCA) 10 µM, or portal plasma (20% or 40%) for 3 h. CTL is the basal activation, i.e., HEK293T cells were transfected with the three plasmids and then exposed to the medium that does not contain the tested condition. Then, cells were lysed and assayed according to the Dual-Luciferase Reporter Assay System (Promega E1910). Firefly and renilla luminescences were quantified using a GloMax 20/20 Luminometer. Firefly luciferase signal was normalized to renilla luciferase signal as an internal control of the transfection efficiency. 

### 2.5. Profiling of Bile Acids

Bile acids were extracted from portal plasma by precipitation with ice-cold methanol. The bile acid species were quantified by high-performance liquid chromatography (UFLC-XR device, Shimadzu, Kyoto, Japan) coupled to tandem mass spectrometry (QTRAP5500 hybrid system, equipped with a Turbo VTM ion source, Sciex) using five deuterated bile acids (d4-CA, d4-GCA, d4-TCA, d4-CDCA, d4-GCDCA) as internal standards, as previously described [27].

### 2.6. Histology and Immunohistochemistry

Formalin-fixed, paraffin-embedded liver or brown adipose tissue sections (4 µm thick) were stained with hematoxylin and eosin (H&E) or used for immunohistochemistry. For the detection of macrophages or neutrophils, liver tissues were incubated with a polyclonal rat anti-mouse F4/80 antibody (1:200, AbD Serotec MCA497G) or a polyclonal rat anti-mouse Ly-6G (1:2000, BD Pharmingen 551459), a polyclonal rabbit anti-rat (1:100, Vector AI-4001) and Envision anti-rabbit-HRP (Dako K4003). For the detection of uncoupling protein 1 (UCP1), brown adipose tissues were incubated with a polyclonal rabbit anti-mouse UCP1 antibody (1:1000, Abcam Ab10983) and Envision anti-rabbit-HRP (Dako K4003). Then, the peroxidase activity was revealed with diaminobenzidine (Dako K3468), and sections were counterstained with hematoxylin. The NAFLD activity score was assessed according to Kleiner et al. [1]. 

### 2.7. RNA Extraction, Reverse Transcription and Real-Time qPCR

RNA was extracted from snap frozen tissues using Trizol. cDNA was then synthetized from 1 µg of RNA and gene expression assessed by quantitative polymerase chain reaction (Rotor-Gene Q, Qiagen, Hilden, Germany). Ribosomal protein L19 (*Rpl19*) was used as a reference gene to normalize the mRNA levels. Primer sequences are listed in Table 1.

### 2.8. Statistics

Statistical analyses were performed using GraphPad Prism 8. Data are presented as mean ± standard deviation. Outliers were removed based on Grubbs’ test. Normality was assessed using the Shapiro–Wilk test. When comparing two groups, an unpaired two tailed *t*-test or Mann–Whitney test was used to calculate significance. When comparing more than two groups, one-way or two-way ANOVA followed by post hoc Bonferroni correction or Kruskal–Wallis followed by Dunn’s multiple comparisons test was used to calculate significance.

## 3. Results

### 3.1. Indirect Activation of TGR5 through Fexaramine

**Figure 1:**
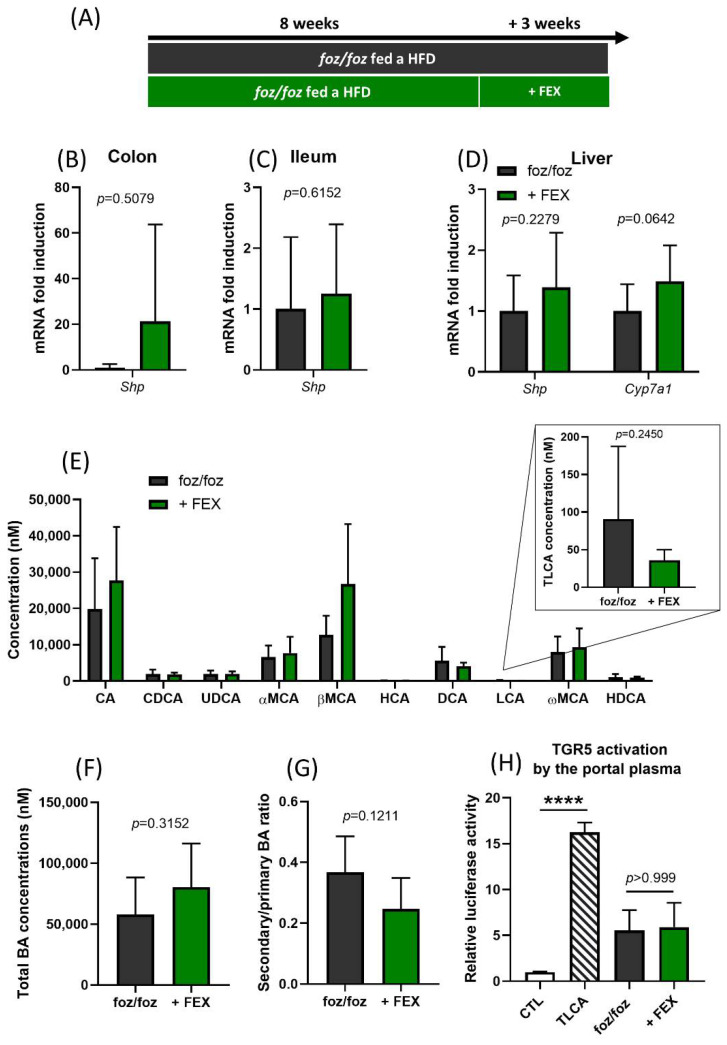
FEX modestly activates FXR signaling in the colon with no effect on TGR5 activation capacity. (**A**) *Foz/foz* mice were fed an HFD for 8 weeks to induce NASH and then submitted for 3 additional weeks to an HFD containing FEX or a plain HFD. (**B**–**D**) Expression of FXR target genes in the colon, ileum and liver of *foz/foz* and *foz/foz* + FEX mice (n = 11–13/group). (**E**) Concentrations of individual bile acid species (sum of conjugated and free bile acids), (**F**) concentration of total bile acids, and (**G**) ratio of secondary to primary bile acids in the portal plasma of *foz/foz* and *foz/foz* + FEX mice (n = 5/group). (**H**) TGR5 activation by the portal plasma of *foz/foz* or *foz/foz* + FEX mice in the cell reporter assay. CTL represents the basal activation and TLCA represents the activation induced by tauro-lithocholic acid, which was used as a positive control. Mean ± SD. Student’s *t* test; and one-way ANOVA followed by post hoc Bonferroni correction. Statistical significance is represented by **** *p* < 0.001.

High-fat diet (HFD)-fed *foz/foz* mice with NASH have an altered bile acid profile with low TGR5 activation capacity [22]. Fexaramine (FEX), a non-absorbed synthetic intestine-restricted FXR agonist, decreases bile acid synthesis and changes the gut microbiota, such as it produces more lithocholic acid (LCA), which is a potent TGR5 activator [12,20]. Thus, *foz/foz* mice were fed an HFD for 8 weeks to induce NASH and then submitted for 3 additional weeks to an HFD containing FEX or a plain HFD (Figure 1A). 

FXR activation was assessed in tissues at the end of the experiment by measuring the expression of FXR target genes. Surprisingly, *Shp* was markedly upregulated in the colon although with a high interindividual variability, but not in the ileum of FEX-treated mice (Figure 1B,C). Other FXR target genes were not affected by treatment (Figure 1B,C and Figure S1A–C). As expected, the treatment with FEX did not affect FXR activation in the liver (Figure 1D), which was in line with the intestinal selectivity of the molecule. In contrast with previous reports, the concentration of individual bile acids, the concentration of total bile acids and the ratio of primary to secondary bile acids were similar in the portal plasma of FEX-treated and untreated *foz/foz* mice (Figure 1E–G). The concentration of the total and tauro-conjugated form of lithocholic (TLCA) was, as well, similar between groups (Figure 1E). In accordance, the TGR5 activation capacity of the portal plasma of *foz/foz* mice was unchanged by FEX (Figure 1H). 

**Figure 2:**
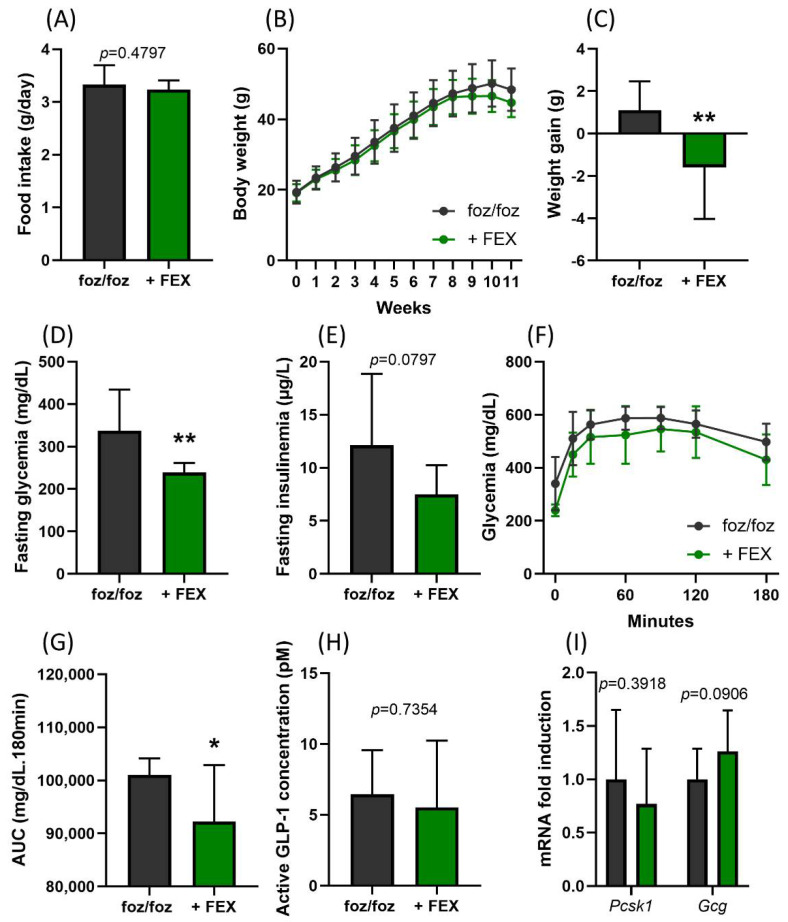
FEX poorly modulates dysmetabolic parameters in mice with NASH. (**A**) Mean food intake, (**B**) body weight evolution, (**C**) body weight gain over the 3 weeks of treatment, (**D**) fasting glycemia and (**E**) fasting insulinemia of *foz/foz* and *foz/foz* + FEX mice. (**F**) Glycemia during the OGTT performed at the end of the experiment and (**G**) AUC for the OGTT. (**H**) Plasmatic active GLP-1 concentrations 30 min after the glucose challenge. (**I**) Gene expression of prohormone convertase 1 (*Pcsk1*) and proglucagon (*Gcg*) in the colon of *foz/foz* and *foz/foz* + FEX mice (n = 11–13/group). Mean ± SD. Two-way ANOVA followed by post hoc Bonferroni correction; and Student’s *t* test. Statistical significance is represented by * *p* < 0.05 and ** *p* < 0.001.

While the presence of FEX in the HFD did not alter food consumption (Figure 2A), three weeks of treatment caused a significant body weight loss (Figure 2B,C) and a reduction in fasting glycemia in HFD-fed *foz/foz* mice (Figure 2D). Fasting insulinemia and glucose tolerance measured by an oral glucose tolerance test also tended to be lower in treated mice (Figure 2E–G). FEX treatment has been shown to increase GLP-1 secretion [12,20]. However, the GLP-1 concentration after the glucose challenge and the gene expression of prohormone convertase 1 (*Pcsk1*) and proglucagon (*Gcg*) in the colon were similar between FEX-treated and untreated *foz/foz* mice (Figure 2H,I), supporting the absence of TGR5 activation. Thus, in our model, FEX treatment did not enhance the amount of LCA or other TGR5 agonists in the enterohepatic cycle. 

### 3.2. Direct Activation of TGR5 by INT-777 and RO5527239

We then used INT-777 and RO5527239 to directly target TGR5 and study the hepatoprotective effects of its activation in the context of NASH. 

#### 3.2.1. INT-777 and RO5527239 Are Well Tolerated and Reactivate TGR5

**Figure 3:**
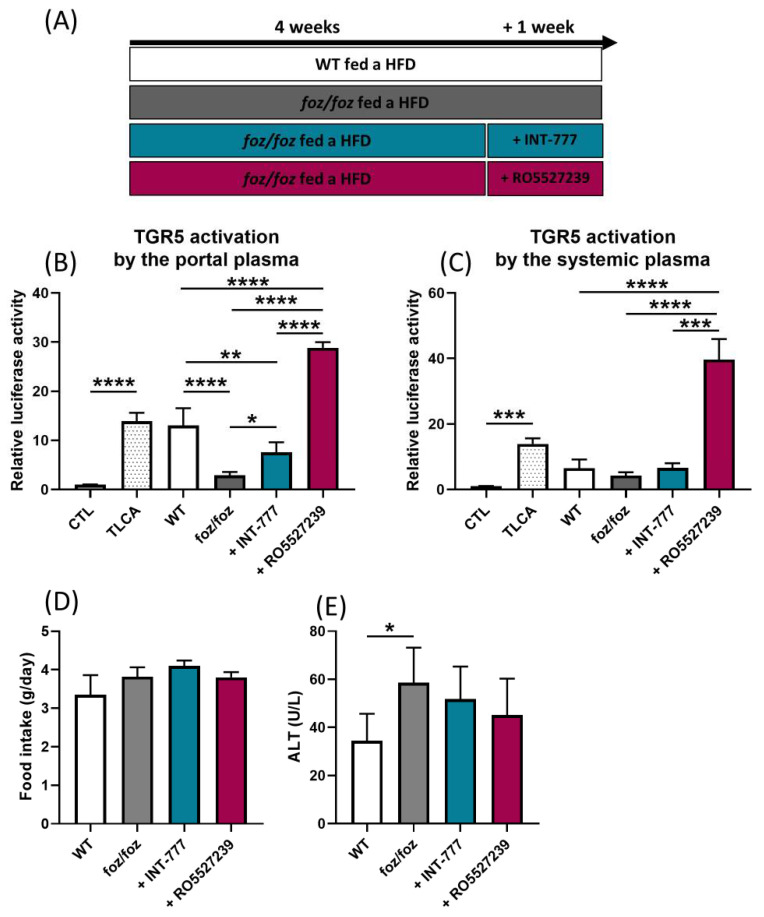
INT-777 and RO5527239 are well tolerated and reactivate TGR5. (**A**) After 4 weeks of HFD, *foz/foz* mice received an HFD containing INT-777, an HFD containing RO5527239 or a plain HFD for one additional week. HFD-fed WT mice were used as controls. (**B**,**C**) TGR5 activation by the portal or systemic plasma of WT, *foz/foz*, *foz/foz* + INT-777 or *foz/foz* + RO5527239 mice (n = 5–7/group) in the cell reporter assay. CTL represents the basal activation and TLCA the activation induced by tauro-lithocholic acid, which was used as a positive control. (**D**) Mean food intake. (**E**) ALT levels in systemic plasma of WT, *foz/foz*, *foz/foz* + INT-777 or *foz/foz* + RO5527239 mice. Mean ± SD. One-way ANOVA followed by post hoc Bonferroni correction. Statistical significance is represented by * *p* < 0.05; ** *p* < 0.01; *** *p* < 0.001 and **** *p* < 0.0001. All *p*-values < 0.05 are represented on the graphs, while *p*-values > 0.05 are not.

We first examined the method of administration and dosing in our model. After 4 weeks of HFD, *foz/foz* mice received an HFD containing INT-777, an HFD containing RO5527239 or plain HFD for one additional week (Figure 3A). The concentration of the compound in the food was adjusted to achieve daily intake of 30 mg/kg of body weight for INT-777 and 10 mg/kg of body weight for RO5527239. HFD-fed WT mice were used as controls (Figure 3A). The TGR5 activation capacity of the portal plasma of *foz/foz* mice was, as previously described [22], significantly lower compared to WT mice (Figure 3B). This difference in TGR5 activation capacity was not observed in systemic plasma (*p* > 0.9999, Figure 3C). INT-777 administration doubled the TGR5 activation capacity of the portal but not of the systemic plasma of *foz/foz* mice (*p* > 0.9999, Figure 3B,C), while RO5527239 administration markedly enhanced the TGR5 agonist load in the portal (>10-fold) as well as in the systemic plasma (>9-fold) of *foz/foz* mice, well above levels observed in WT controls (Figure 3B,C). 

Of note, the daily food intake was similar among the groups, indicating that the presence of either compound did not preclude HFD consumption (Figure 3D, *p* > 0.2971). The body weight of *foz/foz* mice was unchanged by the treatments (data not shown). The plasmatic concentrations of alanine aminotransferases (ALT), reflecting hepatocyte damage and thereby potential toxicity of the compounds, was also unchanged by the treatments in *foz/foz* mice (Figure 3E, *p* > 0.3859).

#### 3.2.2. Effects of Chronic TGR5 Activation on NASH and Associated Metabolic Features

**Figure 4:**
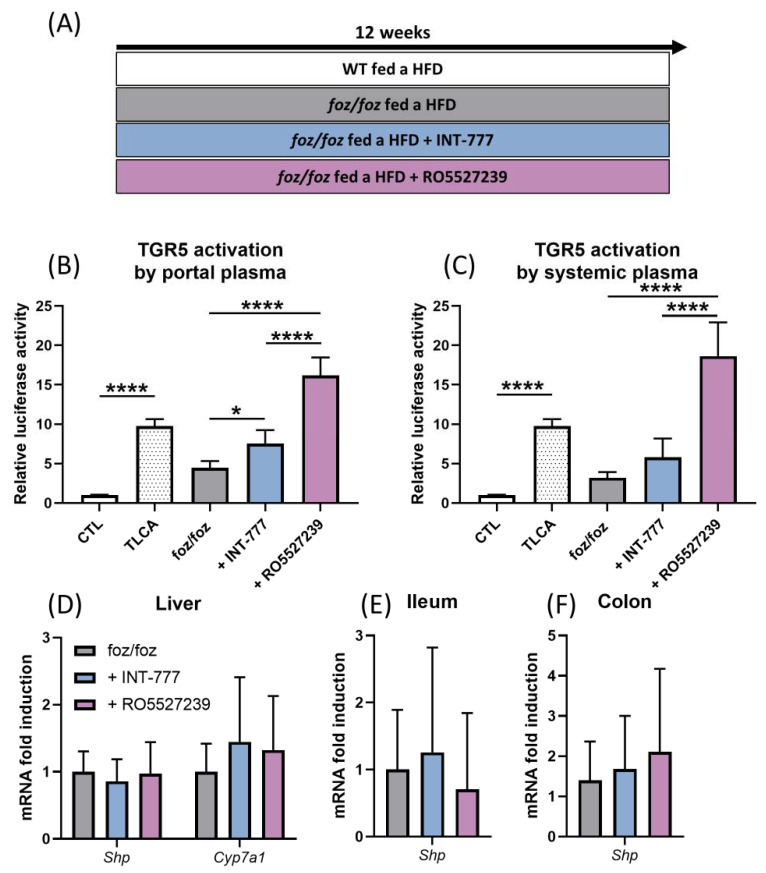
INT-777 and RO5527239 stimulate TGR5 signaling with no effect on FXR signaling. (**A**) *Foz/foz* mice were fed for 12 weeks an HFD supplemented with INT-777 or with RO5527239; then, they were compared to *foz/foz* and WT mice fed an HFD for 12 weeks. (**B**,**C**) TGR5 activation by the portal or systemic plasma of WT, *foz/foz*, *foz/foz* + INT-777 or *foz/foz* + RO5527239 mice in the cell reporter assay. CTL represents the basal activation and TLCA represents the activation induced by tauro-lithocholic acid, which was used as a positive control. (**D**–**F**) Expression of FXR target genes in the liver, ileum and colon of WT, *foz/foz*, *foz/foz* + INT-777 and *foz/foz* + RO5527239 mice (n = 7/group). Mean ± SD. One-way ANOVA followed by post hoc Bonferroni correction. Statistical significance is represented by * *p* < 0.05 and **** *p* < 0.0001. All *p*-values < 0.05 are represented on the graphs, while *p*-values > 0.05 are not.

We next initiated a longer protocol to test the effect of TGR5 reactivation on NASH and associated metabolic features. *Foz/foz* mice were fed for 12 weeks an HFD supplemented with INT-777 or with RO5527239; then, they were compared to *foz/foz* and WT mice fed an HFD for 12 weeks (Figure 4A).

#### 3.2.3. Both INT-777 and RO5527239 Stimulate TGR5 Signaling, with No Effect on FXR Signaling

As in the shorter protocol, the treatment of *foz/foz* mice with INT-777 and more potently with RO5527239 increased the TGR5 activation capacity of the portal plasma (Figure 4B). RO5527239 also significantly increased the TGR5 activation capacity of the systemic plasma of *foz/foz* mice, but INT-777 did not (*p* = 0.7637, Figure 4C). Interestingly, the hepatic gene expression of TGR5 tended to be upregulated by INT-777, but not by RO5527239 (Figure S2A,B). TGR5 activation is known to induce the relaxation of gallbladder smooth muscle cells [28]. The higher volume of gallbladder bile in *foz/foz* mice treated by RO5527239 confirmed higher systemic TGR5 activation compared to the other groups (Figure S2C). While both molecules stimulated TGR5 activation, they did not affect FXR signaling, as shown by similar expression of target gene *Shp* in the liver, ileum and colon of treated and untreated *foz/foz* mice (Figure 4D–F).

#### 3.2.4. Both INT-777 and RO5527239 Improved Glucose Tolerance

**Figure 5:**
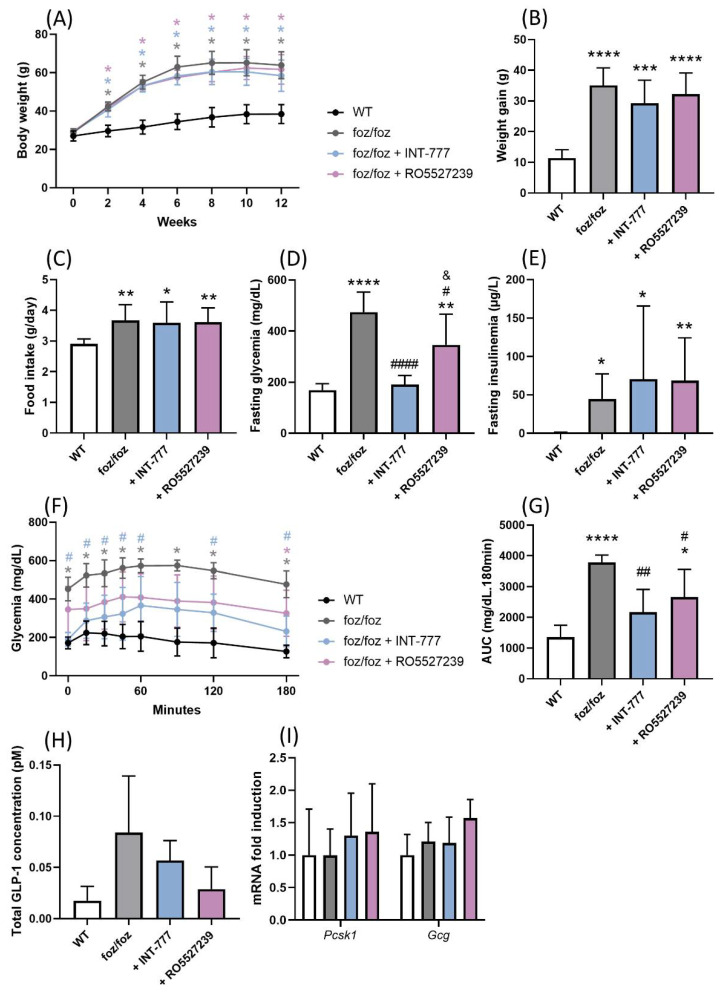
INT-777 and RO5527239 improve glucose tolerance in a GLP-1-independent manner. (**A**) Body weight evolution, (**B**) body weight gain over the 12 weeks, (**C**) mean food intake, (**D**) fasting glycemia and (**E**) fasting insulinemia of WT, *foz/foz*, *foz/foz* + INT-777 and *foz/foz* + RO5527239 mice (n = 7/group). (**F**) Glycemia during the OGTT performed at the end of the experiment and (**G**) AUC for the OGTT. (**H**) Total GLP-1 concentrations in systemic plasma 15 min after a glucose challenge. (**I**) Gene expression of *Pcsk1* and *Gcg* in the colon of WT, *foz/foz*, *foz/foz* + INT-777 and *foz/foz* + RO5527239 mice (n = 7/group). Mean ± SD. One- or two-way ANOVA followed by post hoc Bonferroni correction. Statistical significance is represented by * *p* < 0.05; ** *p* < 0.01; *** *p* < 0.001 and **** *p* < 0.0001 when compared to WT; ^#^ *p* < 0.05; ^##^ *p* < 0.01; and ^####^ *p* < 0.001 when compared to *foz/foz*; and ^&^ *p* < 0.05 when compared to *foz/foz* + INT-777. For (**A**,**F**), colors are used to indicate the corresponding group. All *p*-values < 0.05 are represented on the graphs, while *p*-values > 0.05 are not.

When fed an HFD, *foz/foz* mice developed obesity, fasting hyperglycemia, fasting hyperinsulinemia and glucose intolerance compared to WT mice (Figure 5A–F). Hyperphagia partly explains their phenotype (Figure 5C) [29]. INT-777 or RO5527239 treatment did not alter body weight gain, food intake, fasting insulinemia and insulinemia after glucose challenge in HFD-fed *foz/foz* mice (Figure 5A–C,E and Figure S2D). Nevertheless, INT-777 and, to a lower extend, RO5527239 significantly reduced fasting hyperglycemia and improved glucose tolerance (Figure 5D,F,G). We then investigated whether the TGR5-driven regulation of GLP-1 contributes to the improved glucose tolerance. Surprisingly, the circulating concentration of GLP-1 tended to be higher in *foz/foz* than in WT mice (*p* = 0.087), and was not modified by treatment with INT-777 or RO5527239 (Figure 5H). In accordance, the expression of prohormone convertase 1 (*Pcsk1*) and proglucagon (*Gcg*) genes in colonic mucosae was similar across the groups (Figure 5I). In conclusion, the chronic activation of TGR5 by pharmacological agents improved glucose tolerance in a GLP-1-independent manner in HFD-fed *foz/foz* mice.

#### 3.2.5. RO5527239 Activation of TGR5 in Peripheral Tissues Does Not Rescue Brown Adipose Thermogenesis

**Figure 6:**
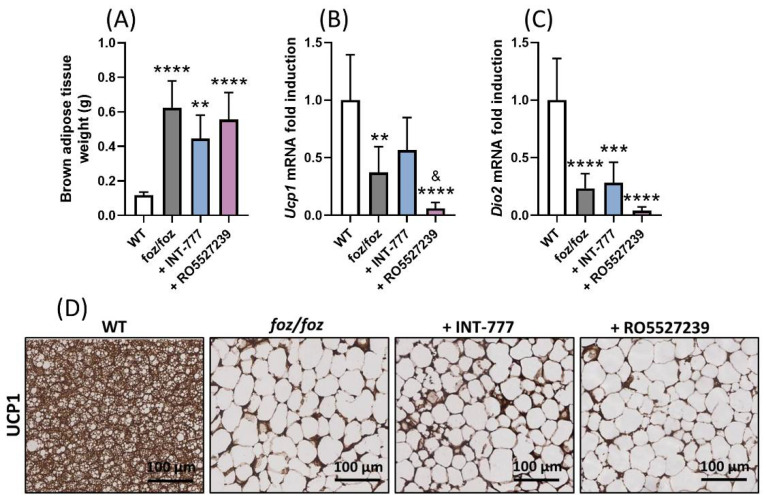
RO5527239-induced activation of TGR5 in peripheral tissues does not rescue brown adipose thermogenesis. (**A**) Brown adipose tissue weight. (**B**,**C**) Gene expression of *Ucp1* and *Dio2*, and (**D**) representative UCP1 staining in the brown adipose tissue of WT, *foz/foz*, *foz/foz* + INT-777 and *foz/foz* + RO5527239 mice (n = 7/group). Mean ± SD. One-way ANOVA followed by post hoc Bonferroni correction. Statistical significance is represented by ** *p* < 0.01; *** *p* < 0.001 and **** *p* < 0.0001 when compared to WT; and ^&^ *p* < 0.05 when compared to *foz/foz* + INT-777. All *p*-values < 0.05 are represented on the graphs, while *p*-values > 0.05 are not.

RO5527239, but not INT-777, increased the TGR5 activation capacity of systemic plasma (Figure 4C). Thus, we anticipate that RO5527239, but not INT-777, would stimulate thermogenesis in the brown adipose tissue of *foz/foz* mice. As previously described [29,30], HFD-fed *foz/foz* mice had an impaired brown adipose thermogenesis, as shown by a higher weight of the tissue and lower uncoupling protein 1 (*Ucp1*) and iodothyronine deiodinase 2 (*Dio2*) gene and UCP1 protein expression compared to their HFD-fed WT controls (Figure 6A–D). Surprisingly, the treatment with RO5527239 did not change weight and lipid content in the brown adipose tissue nor the UCP1 protein expression (Figure 6A,D). RO5527239 did not increase *Ucp1* and *Dio2* mRNA expression (Figure 6B,C). As expected, the brown adipose weight, as well as the gene and protein expressions of *Ucp1* and *Dio2*, were not significantly modified by treatment with INT-777 (Figure 6A–D). In conclusion, the high TGR5 activation capacity of systemic plasma in RO5527239-treated *foz/foz* mice did not stimulate systemic effects such as thermogenesis.

#### 3.2.6. INT-777, but Not RO5527239, Partially Improves NASH

**Figure 7:**
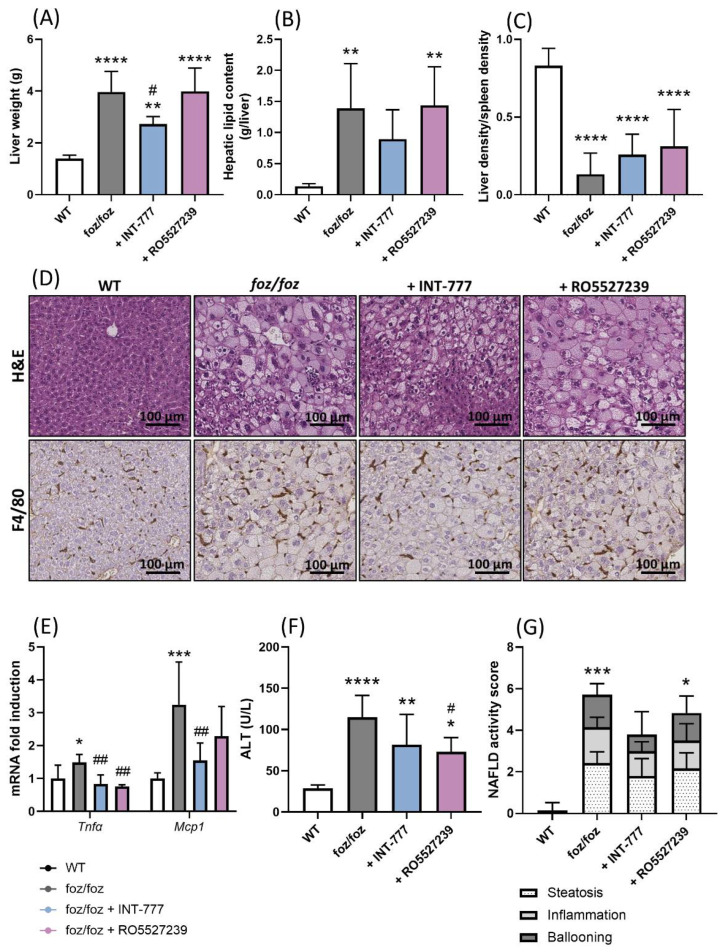
INT-777, but not RO5527239, partially improves NASH. Liver (**A**) weight, (**B**) lipid content, (**C**) density (normalized to spleen density). (**D**) Representative H&E and F4/80 staining of liver sections (bar size: 100 µm). (**E**) Hepatic gene expression of *Tnfα* and *Mcp1*, (**F**) plasmatic ALT levels and (**G**) NAFLD activity score [1] of WT, *foz/foz*, *foz/foz* + INT-777 and *foz/foz* + RO5527239 mice (n = 7/group). Mean ± SD. One-way ANOVA followed by post hoc Bonferroni correction. Statistical significance is represented by * *p* < 0.05; ** *p* < 0.01; *** *p* < 0.001 and **** *p* < 0.0001 when compared to WT; and ^#^ *p* < 0.05; ^##^ *p* < 0.001 when compared to *foz/foz*. All *p*-values < 0.05 are represented on the graphs, while *p*-values > 0.05 are not.

Then, we investigated the effects of the two TGR5 agonists on the liver disease. Compared to WT mice, the liver weight and liver fat content were higher in *foz/foz* mice (Figure 7A,B). Accordingly, liver density, reflecting and inversely correlating with hepatic steatosis [26], was markedly reduced in *foz/foz* mice compared to WT controls (Figure 7C). *Foz/foz* livers showed steatosis, lobular inflammation and ballooning (Figure 7D), as confirmed by higher macrophage infiltration (F4/80 staining), pro-inflammatory gene expressions, ALT levels, and an NAFLD activity score compared to WT livers (Figure 7D–G). Treatment with RO5527239 did not change liver weight, lipid content or density, and it had a limited effect on inflammation and ALT levels (Figure 7A–F). The NAFLD activity score was unchanged by RO5527239 (Figure 7G). In contrast, treatment with INT-777 significantly reduced liver weight, histological steatosis, as well as lipid content and liver density, although not significantly (Figure 7A–D). Hepatic pro-inflammatory genes *Tnfα* and *Mcp1* expression and NAFLD activity score were also reduced by INT-777 (Figure 7E–G).

## 4. Discussion

In this study, pharmacological strategies were used to directly or indirectly activate the potential anti-NASH effect of TGR5. The rationale for such an approach was our earlier demonstration of low TGR5 activation capacity in the enterohepatic cycle of HFD-fed *foz/foz* mice with NASH, with a pathogenic contribution to liver disease [22]. To achieve this, HFD-fed *foz/foz* mice with NASH and associated dysmetabolic features were treated with FEX, INT-777 or RO5527239. First, we showed that the intestine-restricted FXR agonist, FEX, failed to significantly increase FXR activation in the ileal and colonic compartments. In addition, we did not observe a rise in TGR5 ligands as claimed by previous literature reports [12,20]. Accordingly, FEX treatment did not activate TGR5 signaling and did not modulate GLP-1 secretion. Then, we treated HFD-fed *foz/foz* mice with INT-777 or RO5527239, two TGR5 agonists, to directly and potently activate TGR5. While the TGR5 activation capacity was restricted to the portal circulation with INT-777, RO5527239 also amplified the TGR5 activation capacity systemically. Both molecules improved glucose tolerance, although in a GLP-1 independent manner. Despite the differential increase in TGR5 activation capacity by the two agonists, only the modest enterohepatic activation of TGR5 by INT-777, but not the robust activation by RO5527239, partially protected from NASH.

We previously reported the alteration of the enterohepatic bile acid profile in murine NASH, resulting in a low TGR5 and FXR signaling [22]. We also showed that supplementation with the secondary bile acid DCA restored TGR5 and FXR signaling and prevented the development of NASH [22]. In the study of Fang et al., treatment of HFD-fed WT mice with the intestine-restricted FXR agonist FEX (50 mg/kg for 5 weeks) induced FGF15 expression and secretion, and it modulated the bile acid profile [12]. In particular, they observed an increased LCA concentration in the serum [12]. In addition, FEX stimulated thermogenesis and reduced weight gain, inflammation and hepatic steatosis [12]. Pathak et al. also reported that the administration of FEX (50 mg/kg for 6 days) modified the gut microbiota and subsequently increased the production of secondary bile acids (in particular LCA) and TGR5 activation in enteroendocrine L cells [20]. Using *Tgr5*^-/-^ and *Fxr*^-/-^ mice, they showed that the effect depends on FXR targeting and TGR5 activation [20]. In *db/db* mice, another study by Liu et al. showed that FEX (50 mg/kg for 8 weeks) reduced liver steatosis through the activation of the FXR-SHP-CPT1α signaling pathway [31]. Based on these data, FEX was a molecule of choice to investigate the modulation of bile acid profile and its impact on FXR and TGR5 signaling in mice with NASH. However, in our hands, treatment with FEX failed to induce FXR activation to modulate bile acid profile and to increase TGR5 ligands. In the study of Hartmann et al., FEX (100 mg/kg for 8 weeks) poorly affected bile acid metabolism, although it increased FGF15 secretion and improved the gut barrier function and hence protected from alcohol-induced liver injury [32]. Another study by Sorribas et al. described that intestinal FXR activation by FEX (100 mg/kg for 2 weeks) increased the tight junctions protein expression in ileum and reduced bacterial translocation from the gut to the liver in cirrhotic mice [33]. This suggests that the effects of FEX are related to the regulation of the intestinal barrier rather than the incretin effect. However, the poor evidence of gut barrier alteration in the HFD-fed *foz/foz* model (similar intestinal permeability and tight junctions’ expression between HFD-fed *foz/foz* and WT mice; unpublished data and [34]) together with the inefficacy of FEX to reduce liver disease do not support its contribution of this process. In addition, the reports on the effects of intestinal FXR on GLP-1 are conflicting; some authors showed that FEX induces GLP-1 secretion by enteroendocrine L cells [20], while others showed the exact opposite [35,36] or the absence of effect [37]. In HFD-fed *foz/foz* mice, we observed no robust effect of FEX on insulin or on incretin secretion, which was in accordance with no effect on TGR5 activation capacity. Hence, the consequence of intestinal FXR activation on the pathological dysmetabolic context in which it might be of therapeutic value still needs to be refined. 

We then used INT-777 and RO5527239, two synthetic TGR5 agonists: INT-777 moderately increased the portal TGR5 activation capacity in HFD-fed *foz/foz* mice, while RO5527239 markedly enhanced the TGR5 ligand activity of both portal and systemic circulations. INT-777 is a less potent agonist of TGR5 (EC_50_ = 457 nM [6]) than RO5527239 (EC_50_ = 4 nM [18]). In addition, their different chemical structures explain the differences observed between the two molecules: INT-777 is a cholic acid derivative transported as bile acids that undergo enterohepatic cycles [6], while RO5527239 is a non-steroidal molecule [18]. It is poorly taken up by the hepatocytes; thus, it is not secreted in the bile and spills out in the systemic circulation [18]. In addition, the absence of FXR activation in the liver, ileum and colon confirmed the two compounds as specific TGR5 agonists. 

Although being a weaker TGR5 agonist than RO5527239, INT-777 had a beneficial effect on glucose tolerance and on liver disease severity, while RO5527239 had no or limited effect. We anticipated an increased secretion of GLP-1 [6,18,19,38] but, surprisingly, neither INT-777 nor RO5527239 modulated plasmatic concentration of GLP-1 in treated *foz/foz* mice. HFD-fed *foz/foz* mice with fasting hyperglycemia and severe hyperinsulinemia have GLP-1 levels higher than controls, which is in line with the study of Seon et al. reporting that GLP-1 levels correlated with fasting insulinemia in adult with metabolic syndrome [39]. It is possible that further TGR5 stimulation would not further increase GLP-1 synthesis and secretion. Indeed, the high and chronic activation of TGR5, particularly by RO5527239, might lead to a desensitization and a fatigue of the GLP-1 secretagogue mechanism. 

The administration of RO5527239 is associated with increased TGR5 activation capacity in systemic blood. This effect was much weaker with INT-777. We did not measure the concentration of the compounds in portal or systemic blood. Nevertheless, data on TGR5 activation capacity support high portal and systemic concentrations of RO5527239, while the activation of TGR5 by INT-777 in systemic plasma is negligible. This is in accordance with previous reports in rats [40] in which INT-777 concentrations were 1000-fold lower in systemic blood than in bile and liver, supporting the notion that INT-777 remains in the enterohepatic circulation as endogenous bile acids do. Hence, TGR5 activation in tissues and cells outside the enterohepatic cycle could be anticipated, specifically upon RO5527239 treatment. As a confirmation, RO5527239-treated mice showed myorelaxation of the gallbladder musculature. By contrast, and surprisingly, thermogenesis in the brown adipose tissue of *foz/foz* mice was stimulated, although modestly, by the administration of INT-777, while RO5527239 further repressed it. The downregulation of the thermogenic genes by the treatment with a TGR5 agonist is in contradiction with data available in the literature [41]. This suggests that the effect depends on the nature, rather than the potency, of the TGR5 agonist. Alternatively, TGR5 activation in cells of the enterohepatic tissues may lead to the production and/or release of mediators able to modulate signaling pathways (e.g., thermogenesis and energy expenditure) in peripheral tissues (e.g., brown adipose tissue). It will be important to determine in further work whether a beneficial effect on MAFLD depends on TGR5 signaling within and/or outside the enterohepatic tissues.

Overall, in the HFD-fed *foz/foz* mouse model, treatment with either INT-777 or RO5527239 is poorly effective in protecting from NASH, in contrast to the dietary supplementation with the secondary bile acid deoxycholic acid (DCA), as previously reported [22]. We see no correspondence between TGR5 activation capacity (RO5527239 > DCA > INT-777) and hepatoprotective effect (DCA > INT-777 > RO5527239). The difference might reside in mixed TGR5 and FXR activation by DCA but not INT-777 or RO5527239 [23]. Thus, we postulate that joint activation of TGR5 and FXR would (1) provide more protection against NASH and associated dysmetabolic features and (2) limit the side effects observed by selective agonists. Studies comparing dual TGR5 and FXR agonists (e.g., INT-767 [21,42,43,44], BAR502 [45]) to single TGR5 or FXR agonists support this view; this is in accordance with the fact that natural bile acids activate both TGR5 and FXR, even if with different affinities. Next to bile acid receptors, the joint activation of TGR5 and other nuclear receptors such as peroxisome proliferator-activated receptors (PPARs) or liver X receptors (LXRs) would be of interest for the treatment of NASH [46].

To conclude, our data demonstrate the importance of rebalancing bile acid signaling at the enterohepatic rather than at the peripheral level, showing that the enterohepatic agonism of TGR5 has the potential to improve NASH and associated glucose dysmetabolism in a preclinical model of MAFLD.

## Figures and Tables

**Table 1:** Sequences of the primers used for qPCR.

Gene	Forward	Reverse
*Asbt*	TGGATAGATGGCGACATGGA	GGCAAGCAGTGTGGAGCAA
*Bsep*	CTGCCAAGGATGCTAATGCA	CGATGGCTACCCTTTGCTTCT
*Cyp7a1*	AGCAACTAAACAACCTGCCAGTACTA	GTCCGGATATTCAAGGATGCA
*Dio2*	TCGGTCATTCTGCTCAAGCA	AGCATGCGCCTCCACTCT
*Fabp6*	GTGGAAAGTAGACCGGAACGA	GGAAGCAGCAGAAGCTTGGT
*Fgf15*	GACCAAAACGAACGAAATTTGTT	ACGTCCTTGATGGCAATCG
*Fxr*	AGGAGCCCCTGCTTGATGT	GCGGGTTCTCAGGCTGGTA
*Gcg*	CATTCACCAGCGACTACAGCAA	TCATCAACCACTGCACAAAATCT
*Il1β*	AGTTGACGGACCCCAAAAGA	GGACAGCCCAGGTCAAAGG
*Mdr2*	CCACAGATGCTGCGCAAGT	TGGCTGTGTTCTGTGCAATTAAA
*Mcp1*	CCACTCACCTGCTGCTACTCAT	CTGCTGGTGATCCTCTTGT
*Ntcp*	CTTGCGCCATAGGGATCTTC	TGCCTGCCTTGAGGACGTA
*Oatp1a1*	GCCAACGCAAGATCCAACAGAGTG	TCGGGCCAACAATCTTCCCCAT
*Oatp1a4*	CAAGCTTTCTCCCTGCACTCTT	TCCTTCGCAGTGAGCTTCATT
*Ostα*	CCGTCAAGCCAAGATGCAT	CAAGCACCTGGAACAGAGCAA
*Ostβ*	CCGGGGGAACCTGAGTAGAA	GTTATGGGGCGTTATGGGGT
*Pcsk1*	TGTACTGCTTTCGCCTTCTTTT	CGCCGCCCATTCATTAACA
*Rpl19*	GAAGGTCAAAGGGAATGTGTTCA	CCTTGTCTGCCTTCAGCTTGT
*Shp*	AGGGTAGAGGCCATGAGGAG	ACGATCCTCTTCAACCCAGA
*Tgr5*	GGCCTGGAACTCTGTTATCG	GTCCCTCTTGGCTCTTCCTC
*Tnfα*	GTGCCTATGTCTCAGCCTCTT	GCTCATACCAGGGTTTGAGCT
*Ucp1*	CGTACCAAGCTGTGCGATGT	GAAGCCACAAACCCTTTGAAAA

## Supplementary Materials

The following supporting information can be downloaded at: https://www.mdpi.com/article/10.3390/nu14132707/s1. Figure S1: Gene expression of FXR, of its target genes and of TGR5 in the colon (A), ileum (B) and liver (C) of foz/foz and foz/foz + FEX mice, Figure S2: Gene expression of TGR5 in the colon (A) and liver (B) of WT, foz/foz, foz/foz + INT-777 and foz/foz + RO5527239 mice. (C) Volume of bile in the gallbladder of WT, foz/foz, foz/foz + INT-777 and foz/foz + RO5527239 mice. (D) Insulinemia 15 min after the administration of glucose to fasted WT, foz/foz, foz/foz + INT-777 and foz/foz + RO5527239 mice.

## Abbreviations

ALT: alanine aminotransferase; CA, cholic acid; CDCA, chenodeoxycholic acid; DCA, deoxycholic acid; DIO2, iodothyronine deiodinase 2; FEX, fexaramine; FGF15, fibroblast growth factor 15; FXR, Farnesoid X receptor; GCG, proglucagon; GLP-1, glucagon-like peptide-1; HCA, hyocholic acid; HDCA, hyodeoxycholic acid; HFD, high-fat diet; LCA, lithocholic acid; MAFLD, metabolic dysfunction-associated fatty liver disease; MCA, muricholic acid; MCP1, monocyte chemoattractant protein-1; NAFLD, non-alcoholic fatty liver disease; NASH, non-alcoholic steatohepatitis; OGTT, oral glucose tolerance test; PCSK1, prohormone convertase 1; SHP, small heterodimer protein; TGR5, Takeda G-protein coupled receptor 5; TLCA, tauro-lithocholic acid; TNFα, tumor necrosis factor α; UCP1, uncoupling protein 1; UDCA, ursodeoxycholic acid; WT, wildtype.
