# Supplementary material for: Enterohepatic Takeda G-Protein Coupled Receptor 5 Agonism in Metabolic Dysfunction-Associated Fatty Liver Disease and Related Glucose Dysmetabolism
Source: Nutrients. 2022 Jun 29;14(13):2707. doi: 10.3390/nu14132707 (PMC9268629; doi:10.3390/nu14132707)
Supplement: Supplementary file 1 [file nutrients-14-02707-s001.zip › nutrients-1740984-supplementary.pdf]

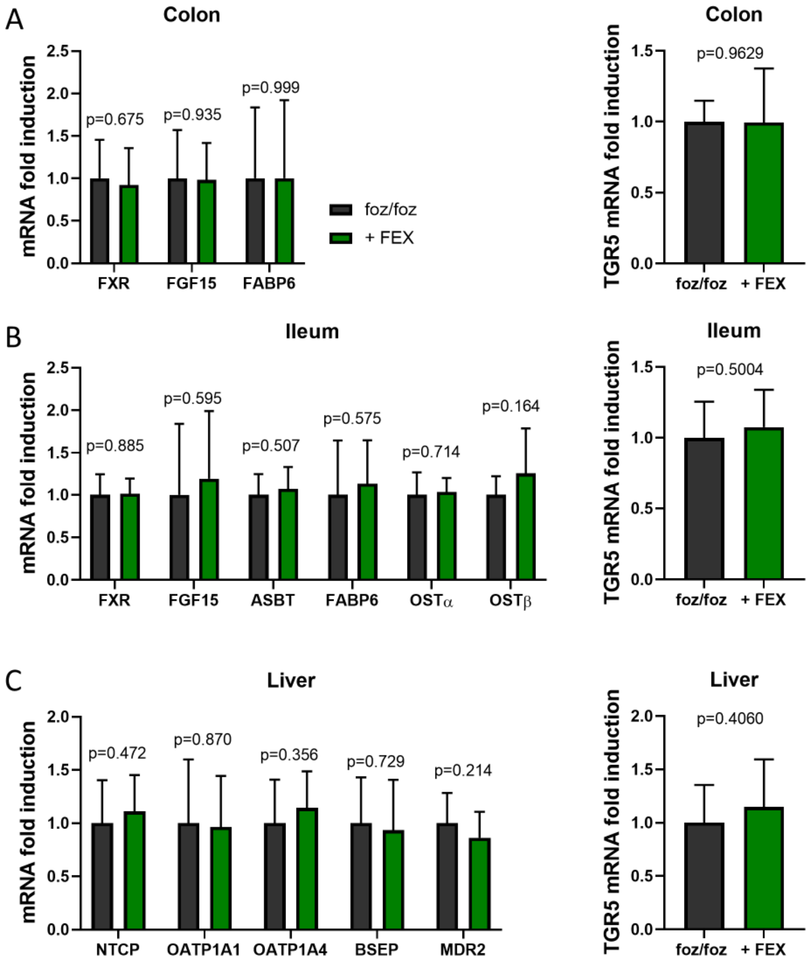

.

Figure S1. Gene expression of FXR, of its target genes and of TGR5 in the colon (A), ileum (B) and liver (C) of *foz/foz* and *foz/foz* + FEX mice. Mean ± SD. Student t test.

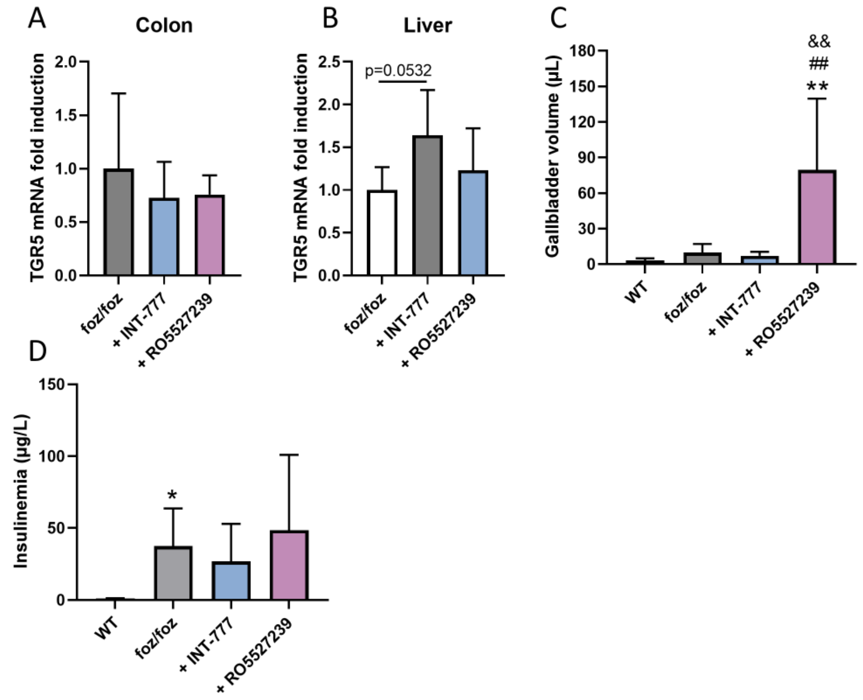

.

Figure S2. Gene expression of TGR5 in the colon (A) and liver (B) of WT, *foz/foz*, *foz/foz* + INT-777 and *foz/foz* + RO5527239 mice. (C) Volume of bile in the gallbladder of WT, *foz/foz*, *foz/foz* + INT-777 and *foz/foz* + RO5527239 mice. (D) Insulinemia 15 minutes after the administration of glucose to fasted WT, *foz/foz*, *foz/foz* + INT-777 and *foz/foz* + RO5527239 mice. Mean ± standard deviation. One-way ANOVA followed by post-hoc Bonferroni correction. Statistical significance is represented by **$p<0.001$ when compared to WT; ##$p<0.001$; when compared to *foz/foz*; and &&$p<0.01$ when compared to *foz/foz* + INT-777. All p-values < 0.05 are represented on the graphs. Statistical significance is represented by *$p<0.05$ and **$p<0.01$ when compared to WT; ##$p<0.001$; when compared to foz/foz; and &&$p<0.01$ when compared to foz/foz + INT-777. All p-values < 0.05 are represented on the graphs.
